# Psychometric Properties of the Chinese Version of the Perceived Stress Scale in Policewomen

**DOI:** 10.1371/journal.pone.0028610

**Published:** 2011-12-02

**Authors:** Zhen Wang, Jue Chen, Jennifer E. Boyd, Haiyin Zhang, Xiuzhen Jia, Jianyin Qiu, Zeping Xiao

**Affiliations:** 1 Shanghai Mental Health Center, Shanghai Jiao Tong University School of Medicine, Shanghai, China; 2 Department of Psychiatry, University of California San Francisco, and San Francisco Veterans Affairs Medical Center, San Francisco, California, United States of America; University of Illinois at Chicago, United States of America

## Abstract

**Background:**

The 10-item Perceived Stress Scale (PSS-10) is one of most widely used instruments to measure a global level of perceived stress in a range of clinical and research settings. This study was conducted to examine the psychometric properties of the Simplified Chinese version of the PSS-10 in policewomen.

**Methodology:**

A total of 240 policewomen were recruited in this study. The Simplified Chinese versions of the PSS-10, the Beck Depression Inventory Revised (BDI-II), and the Beck Anxiety Inventory (BAI) were administered to all participants, and 36 of the participants were re-tested two weeks after the initial testing.

**Principal Findings:**

The overall Cronbach's alpha was 0.86, and the test–retest reliability coefficient was 0.68. Exploratory Factor Analysis (EFA) yielded 2 factors with eigenvalues of 4.76 and 1.48, accounting for 62.41% of variance. Factor 1 consisted of 6 items representing “negative feelings”; whereas Factor 2 consisted of 4 items representing “positive feelings”. The item loadings ranged from 0.72 to 0.83. The Confirmatory factor analysis (CFA) indicated a very good fit of this two-factor model to this sample. The PSS-10 significantly correlated with both BDI-II and BAI, indicating an acceptable concurrent validity.

**Conclusions:**

The Simplified Chinese version of the PSS-10 demonstrated adequate psychometric properties for evaluating stress levels. The results support its use among the Chinese population.

## Introduction

Psychological stress occurs when an individual perceives that environmental demands tax or exceed his or her adaptive capacity [1]. A number of studies have provided evidence that psychological stress is associated with depression, anxiety, cardiovascular disease, cancer, and other adverse health outcomes [2], [3], [4], [5], [6], [7]. Lazarus proposed that for an event or situation to be considered stressful, it must be perceived as stressful via perceptual processes [8]. Therefore, it is important to assess the perceived stress level when evaluating the impact of psychological stress on health. The Perceived Stress Scale (PSS) [9] has become one of the most widely used instruments for measuring nonspecific perceived stress.

The PSS is a self-report measure developed by Cohen and his colleagues in 1983 [9]. The 10-items version of PSS (PSS-10) has demonstrated adequate reliability and validity [9], [10]. It has been used in various clinical settings, cultures and populations, and has been translated into many languages including Arabic, Chinese (traditional), Japanese, Portuguese and Thai [11], [12], [13], [14], [15]. The Traditional Chinese version of the PSS-10 has been used in Hong Kong and Taiwan, and has shown acceptable reliability and validity [11], [16]. The Chinese character system used in mainland China is Simplified Chinese. The majority of the world's Chinese-speaking population use Simplified Chinese, but the psychometric properties of the Simplified Chinese version of the PSS have yet to be examined.

Police work is widely regarded as one of the most stressful occupations [17], [18], [19]. As a part of a project which investigated the impact of stress on health of policewomen who were on duty at the Shanghai World Expo, we evaluated the reliability, validity and factor structure of the Simplified Chinese version of PSS-10 in a sample of policewomen.

## Methods

### Ethics Statement

This research protocol was approved by the Ethics Committee of the Shanghai Mental Health Center, and written informed consent has been obtained from all study participants.

### Participants

Participants were policewomen who were employed to carry out health and safety checks at the Shanghai World Expo in July 2010. These policewomen were police academy graduates who trained for this specific role for 8 hours per day for a period of six months. There were a total of 9 squadrons of policewomen on duty during the study period. Each squadron contains about 80 to 90 policewomen. Three of these squadrons were randomly selected to receive this survey We did not recruit male police officers because it was outside the scope of the larger study of which this study is a part. Study procedures were explained in detail to all the policewomen in the three selected squadrons, and those who provided written informed consent were included in the study. In total, 260 policewomen were invited to participate, and 245(94.5%) of them agreed to take part in this study. Of these, 240 policewomen completed all of the study measures. All participants were asked to complete a set of self-administered questionnaires in a designated room, and then return the completed questionnaires to an on-site research assistant. Thirty-six participants were randomly selected to complete all the questionnaires again two weeks after the initial testing.

### Instruments

#### Perceived Stress Scale-10 (PSS-10)

The PSS-10 [10] measures the degree to which one perceives aspects of one's life as uncontrollable, unpredictable, and overloading. Participants are asked to respond to each question on a 5-point Likert scale ranging from 0 (never) to 4 (very often), indicating how often they have felt or thought a certain way within the past month. Scores can range from 0 to 40, with higher composite scores indicative of greater perceived stress. The PSS-10 has demonstrated good reliability and validity, with Cronbach's alphas ranging from 0.78 to 0.91 and test-retest reliability coefficients ranging from 0.55 to 0.85 [9], [10], [20].

The PSS-10 was translated from the original English version into Simplified Chinese by two native Chinese-speaking psychiatrists working independently of each other and, in a second step, they agreed on a final common translation. After that, the Simplified Chinese version of PSS was back-translated by an English-Chinese bilingual psychologist who had no knowledge of the wording of the original English version of the PSS. The two English versions were then compared item-by-item and minor discrepancies were addressed and corrected in the Simplified Chinese version by a consensus of these translators. Fifteen medical school postgraduate students were asked to complete the Simplified Chinese version of PSS-10 as a pilot study. Further corrections to the translation were completed based on the results of this pilot study.

#### The Beck Depression Inventory Revised (BDI-II) [21]

The BDI-II is a 21-item self-administered instrument designed to assess the severity of depression symptoms over the preceding week. Each item is assigned a score of 0–3, with 3 indicating the most severe symptoms. A cumulative score is determined by adding the scores of the individual items. The total score can range from 0 to 63. The BDI-II is a reliable and well-validated measure in screening for depression symptoms in adults, with Cronbach's alphas ranging from 0.73 to 0.95, and the Simplified Chinese version has been widely used in China [22].

#### The Beck Anxiety Inventory (BAI) [23]

The BAI is a 21-question 4-point self-report inventory that is used for measuring the severity of an individual's anxiety. Possible scores range between 0 and 63. Increasing scores indicate increasing intensity of anxiety symptoms. The BAI is a widely accepted instrument with high internal consistency (Cronbachs α = 0.92), a test-retest reliability over one week of 0.75, and it has been shown to have good validity [23]. The Simplified Chinese version of the BAI has similar psychometric properties [24].

### Analytic Strategy

Descriptive statistics for continuous variables were reported as means and standard deviations. The internal consistency reliability and the test-retest reliability of the Simplified Chinese version of the PSS-10 were assessed using Cronbach's alpha coefficient and Spearman's correlation coefficient, respectively.

To analyze the construct structure of the Simplified Chinese version of the PSS-10, the sample was randomly split into two halves. With the first half, we conducted an exploratory factor analysis (EFA), and with the second half, we conducted a confirmatory factor analysis (CFA). An independent t-test and chi-square test were employed to compare the samples' characteristics. The EFA was performed with principal component and Varimax rotation. The sample adequacy was assessed by the Kaiser-Meyer-Olkin (KMO) measure of sampling adequacy. The CFA was performed to assess the goodness-of- fit of the factor structure extracted from the EFA.

Evidence of concurrent validity was assessed by Pearson correlation between the PSS-10 and symptoms of depression and anxiety measured by BDI-II and BAI respectively.

The CFA analyses were performed with AMOS 7.0 (SPSS Inc., Chicago, IL, USA). All the other analyses were performed with SPSS version 17.0 (SPSS Inc., Chicago, IL, USA). All analyses employed a significance level of 0.05.

## Results

### Participant characteristics

The mean age of the participants was 21.1 years with a standard deviation of 1.4 years. On average, they have 14.5 years of education (SD = 1.3). The majority (97%) of the participants were single. The participants were young and unmarried because they were in squadrons composed entirely of new recruits. The mean score of the PSS-10 reported in this sample was 15.2 (SD = 5.6).

### Exploratory factor analysis (EFA)

All of the diagnostic tests indicated the adequacy of proceeding with factor analysis. Specifically, Bartlett's test of sphericity was statistically significant (p<0.0001), and the Kaiser-Meyer-Olkin (KMO) value was 0.87. The EFA showed that a rotated factor solution for the PSS-10 (Table 1) contained two factors with eigenvalues greater than 1.0, which accounted for 62.41% of the variance. Factor 1 consisted of 6 items representing “negative feelings” (Items 1, 2, 3, 6, 9, and 10) and accounting for 47.61% of the variance; whereas Factor 2 consisted of 4 items representing “positive feelings” (Items 4, 5, 7, and 8) and accounting for 14.80% of the variance. Item loadings ranged from 0.72 to 0.83 (Table 1).

**Table 1:** Exploratory factor analysis and reliability coefficients of PSS-10 (n = 120).

PSS Items	Factor loadings	
	Factor 1	Factor 2
1. In the last month, how often have you been upset because of something that happened unexpectedly?	**0.74**	0.12
2. In the last month, how often have you unable to control the important things in your life?	**0.76**	0.20
3. In the last month, how often have you felt nervous and ‘stressed’?	**0.83**	0.17
4. In the last month, how often have you confident about your ability to handle your personal problems?	0.24	**0.77**
5. In the last month, how often have you felt that things were going your way?	0.13	**0.75**
6. In the last month, how often have you found that you could not cope with all the things that you had to do?	**0.77**	0.16
7. In the last month, how often have you been able to control irritations in your life?	0.09	**0.76**
8. In the last month, how often have you felt that you were on top of things?	0.38	**0.72**
9. In the last month, how often have you been angered because of things that were outside of your control?	**0.75**	0.25
10. In the last month, how often have you felt difficulties were piling up so high that you could not overcome them?	**0.74**	0.26
**Eigenvalue**	4.76	1.48
**% variance**	47.61	14.80
**Cronbach's alpha coefficient**	0.87	0.77

### Confirmatory factor analysis (CFA)

The CFA was used to determine the goodness-of-fit of the previously identified two-factor model. The maximum-likelihood estimation (MLE) method was used to test the covariance matrix to determine how well the model fit the sample data. The CFA goodness-of-fit measures showed that the two-factor solution was adequate: χ^2^ = 43.640 (df = 34, p<0.124); Goodness-of-Fit Index (GFI) = 0.936; Normed Fit Index (NFI) = 0.919; Comparative Fit Index (CFI) = 0.980. Root Mean Square Residual (RMR) = 0.028; Root Mean square Error of Approximation (RMSEA) = 0.048. The standardized regression coefficients ranged from 0.67 to 0.82 for Factor 1 and from 0.59 to 0.80 for Factor 2. The between-factors correlation was 0.62.

### Reliability

Cronbach's alpha for assessing the internal consistency reliability of the PSS-10 was 0.86 for the whole scale, 0.87 for Factor 1, and 0.77 for Factor 2. The two-week test-retest reliability of the PSS-10 was 0.68 for the whole scale, 0.72 for Factor 1, and 0.63 for Factor 2.

### Concurrent Validity

Correlations between the PSS, the BDI-II, and the BAI were calculated (Table 2). As expected, both the latter scales correlated positively with the PSS. The correlation coefficient between the factors and total score of C-PSS-10 and other two scales ranged from 0.36 to 0.67.

**Table 2:** Correlations of PSS-10 to depression (BDI-II) and anxiety (BAI).

	PSS (total)	Factor 1	Factor 2	BDI-II
Factor 1	0.93			
Factor 2	0.76	0.47		
BDI-II	0.67	0.63	0.49	
BAI	0.58	0.59	0.36	0.72

## Discussion

To the authors' knowledge, this is the first study designed to evaluate the reliability and validity of the Simplified Chinese version of the PSS-10 scale. Overall, the psychometric data presented in this study support the conclusion that the Simplified Chinese version of the PSS-10 (C-PSS-10) has adequate psychometric properties.

The overall Cronbach's alpha of the Simplified Chinese version of PSS-10 was 0.86 in this sample. This value is in accord with findings from other studies of different language versions, where reliability coefficients ranged from 0.78–0.91 [10], [11], [12], [13], [14], [15], [20], [25]. The two-week test-retest reliability of C-PSS-10 was 0.68, which is acceptable when compared with the original findings that the test-retest reliability was 0.85 in the college sample after 2 days and 0.55 in the community sample after 6 weeks [9].

Previous studies have shown that the PSS-10 has concurrent validity with a number of other measures including the State Trait Anxiety Inventory (STAI) and the Beck Depression Inventory (BDI) [15], [26]. In the current study, the Simplified Chinese version of PSS-10 was also found to be significantly and moderately positively correlated with measures of anxiety and depression (r = 0.58 for BAI and 0.67 for BDI; p<0.001), and thus the construct validity of this scale was confirmed. These results also indicate that psychological stress is associated with mental health issues.

With regard to the PSS-10′s factor structure, researchers have found that it has 2 related latent factors [10], [14], [15], [25], representing positive and negative feelings. In the present study, the EFA yielded the same result as those found in other language versions [10], [14], [15]. In Cohen's original analysis, two factors yielded eigenvalues of 3.4 and 1.4, which accounted for 48.9% and 14.5% of the variance respectively [10]. In the present study, the Simplified Chinese version of the PSS-10 yielded eigenvalues of 4.76 and 1.48, and accounted for 47.61% and 14.80% of the variance respectively. Concerning item loadings, item 8 had high loadings (>0.3) on the other factors in the present study. Similar results have been reported in other studies [15]. The CFA demonstrated a relatively better goodness-of-fit for the two-factor solution model for the Simplified Chinese version compared to the original version, which found that the PSS-10 revealed an adequate two-factor solution: goodness of fit index = 0.926, Root Mean Square Residual = 0.039, Comparative Fit Index = 0.931 [10]. Although we confirmed the two-factor model of PSS-10, we do not recommend using two separate sub-scales clinically. The PSS-10′s authors suggested that any distinction between these factors is irrelevant [10], and another study also suggested to use the full scale as a whole to evaluate perceived stress level [14].

There are several limitations to this study that should be noted. First, police have a very special occupation, which is full of stressful events in daily work. We can see this stress in our data because the sample's average score on the C-PSS-10 was relatively high compared to the community residents used in the original norms [10]. Additionally, we recruited only female police officers in this study, and all the participants were relatively young. Thus, the characteristics of this sample may limit its generalizability of the results to other populations. However, it is worth noting that according to studies of the English version, the PSS is not a specific-population- dependent instrument. Similar psychometric properties have been found across a variety of different sub-populations in different locations [10], [20], [27]. The Traditional Chinese version of PSS has also been found to have similar psychometric properties in two different sub-groups in Hong Kong [11], [28]. Therefore, on the basis of these findings we expect that the Simplified Chinese version could be validly used in a broader range of Chinese-speaking populations as well. Second, because of the cross-sectional nature of this study, the predictive validity of this scale could not be confirmed. Third, this study was not able to evaluate the discriminant validity of the C-PSS-10 and further study in that area is needed. Fourth, like other studies using similar methodologies [15], [26], we were unable to determine the extent to which our concurrent validity findings represent an overlap between the constructs of stress, depression, and anxiety such that the PSS could be considered to be a “proxy measure” for depression or anxiety. Fifth, it was outside the scope of our study to determine whether additional items might be necessary to capture additional culturally-relevant aspects of reactions to stress. Future studies might examine each of these issues.

In conclusion, the results of this study suggest that the Simplified Chinese version of the PSS-10 is an instrument with adequate psychometric properties. Therefore, the Simplified Chinese version of PSS-10 can be a very useful instrument to measure psychological stress among Chinese-speaking populations in China and elsewhere in the world.

## References

[1] Cohen S, Kessler RC, Gordon LU (1995). Measuring stress: a guide for health and social scientists..

[2] Apostolo JL, Figueiredo MH, Mendes AC, Rodrigues MA (2011). Depression, anxiety and stress in primary health care users.. Rev Lat Am Enfermagem.

[3] Hsieh YH, Hsu CY, Liu CY, Huang TL (2011). The Levels of Stress and Depression among Interns and Clerks in Three Medical Centers in Taiwan - A Cross-sectional Study.. Chang Gung Med J.

[4] Lombard JH (2010). Depression, psychological stress, vascular dysfunction, and cardiovascular disease: thinking outside the barrel.. J Appl Physiol.

[5] Danzer SC (2011). Depression, stress, epilepsy and adult neurogenesis.. Exp Neurol.

[6] Sims R, Gordon S, Garcia W, Clark E, Monye D (2008). Perceived stress and eating behaviors in a community-based sample of African Americans.. Eat Behav.

[7] Cohen S, Janicki-Deverts D, Miller GE (2007). Psychological stress and disease.. JAMA.

[8] Lazarus RS, Folkman S (1984). Stress, appraisal, and coping..

[9] Cohen S, Kamarck T, Mermelstein R (1983). A global measure of perceived stress.. J Health Soc Behav.

[10] Cohen S, Williamson G, Spacapan S, Oskamp S (1988). Perceived stress in a probability sample of the United States.. The Social Psychology of Health: Claremont Symposium on Applied Social Psychology.

[11] Leung DY, Lam TH, Chan SS (2010). Three versions of Perceived Stress Scale: validation in a sample of Chinese cardiac patients who smoke.. BMC Public Health.

[12] Chaaya M, Osman H, Naassan G, Mahfoud Z (2010). Validation of the Arabic version of the Cohen Perceived Stress Scale (PSS-10) among pregnant and postpartum women.. BMC Psychiatry.

[13] Mimura C, Griffiths P (2008). A Japanese version of the Perceived Stress Scale: cross-cultural translation and equivalence assessment.. BMC Psychiatry.

[14] Reis RS, Hino AA, Anez CR (2010). Perceived stress scale: reliability and validity study in Brazil.. J Health Psychol.

[15] Wongpakaran N, Wongpakaran T (2010). The Thai version of the PSS-10: An Investigation of its psychometric properties.. Biopsychosoc Med.

[16] Chen CH, Tseng YF, Chou FH, Wang SY (2000). Effects of support group intervention in postnatally distressed women. A controlled study in Taiwan.. J Psychosom Res.

[17] Violanti JM, Burchfiel CM, Miller DB, Andrew ME, Dorn J (2006). The Buffalo Cardio-Metabolic Occupational Police Stress (BCOPS) pilot study: methods and participant characteristics.. Ann Epidemiol.

[18] Wang Z, Inslicht SS, Metzler TJ, Henn-Haase C, McCaslin SE (2010). A prospective study of predictors of depression symptoms in police.. Psychiatry Res.

[19] Morash M, Kwak D, Haarr R (2006). Gender differences in the predictors of police stress.. Policing: An International Journal of Police Strategies and Management.

[20] Mitchell AM, Crane PA, Kim Y (2008). Perceived stress in survivors of suicide: psychometric properties of the Perceived Stress Scale.. Res Nurs Health.

[21] Beck AT, Steer RA, Brown GK, Antonio San (1996). BDI-II, Beck depression inventory: manual.. Tex.

[22] Wang Z, Yuan C, Huang J, Li Z, Chen J (2011). Reliability and validity of the Chinese version of Beck Depression Inventory-II among depression patients.. Chinese Ment Health J.

[23] Beck AT, Epstein N, Brown G, Steer RA (1988). An inventory for measuring clinical anxiety: psychometric properties.. J Consult Clin Psychol.

[24] Cheng K, Wong C, Wong K (2002). A Study of Psychometric Properties, Normative Scores and Factor Structure of Beck Anxiety Inventory Chinese Version.. Chinese J Clin Psychology.

[25] Roberti JW, Harrington LN, Storch EA (2006). Further Psychometric Support for the 10-Item Version of the Perceived Stress Scale.. Journal of College Counseling.

[26] Stauder A, Thege BK (2006). Summary on the validity study of the Hungarian version of the Perceived Stress Scale.. http://www.psy.cmu.edu/~scohen/PSS_Hungarian_validity_info.pdf.

[27] Sharp LK, Kimmel LG, Kee R, Saltoun C, Chang CH (2007). Assessing the Perceived Stress Scale for African American adults with asthma and low literacy.. J Asthma.

[28] Yu R, Ho S (2010). Psychometric Evaluation of the Perceived Stress Scale in Early Postmenopausal Chinese Women.. Psychology.

